# Somatic copy number alterations are predictive of progression-free survival in patients with lung adenocarcinoma undergoing radiotherapy

**DOI:** 10.20892/j.issn.2095-3941.2020.0728

**Published:** 2021-08-27

**Authors:** Fan Kou, Lei Wu, Yan Guo, Bailu Zhang, Baihui Li, Ziqi Huang, Xiubao Ren, Lili Yang

**Affiliations:** 1 Department of Immunology, Tianjin Medical University Cancer Institute and Hospital, National Clinical Research Center for Cancer, Key Laboratory of Cancer Prevention and Therapy, Key Laboratory of Cancer Immunology and Biotherapy, Tianjin 300060, China; 2 Department of Biotherapy, Tianjin Medical University Cancer Institute and Hospital, National Clinical Research Center for Cancer, Tianjin 300060, China

**Keywords:** SCNA, radiotherapy, lung cancer, progression-free survival, immune infiltration

## Abstract

**Objective:**

Lung cancer is the most common cause of cancer-related deaths worldwide. Somatic copy number alterations (SCNAs) have been used to predict responses to therapies in many cancers, including lung cancer. However, little is known about whether they are predictive of radiotherapy outcomes. We aimed to understand the prognostic value and biological functions of SCNAs.

**Methods:**

We analyzed the correlation between SCNAs and clinical outcomes in The Cancer Genome Atlas data for 486 patients with non-small cell lung cancer who received radiotherapy. Gene set enrichment analyses were performed to investigate the potential mechanisms underlying the roles of SCNAs in the radiotherapy response. Our results were validated in 20 patients with lung adenocarcinoma (LUAD) receiving radiotherapy.

**Results:**

SCNAs were a better predictor of progression-free survival (PFS) in LUAD (*P* = 0.024) than in lung squamous carcinoma (*P* = 0.18) in patients treated with radiotherapy. Univariate and multivariate regression analyses revealed the superiority of SCNAs in predicting PFS in patients with LUAD. Patients with stage I cancer and low SCNA levels had longer PFS than those with high SCNA levels (*P* = 0.022). Our prognostic nomogram also showed that combining SCNAs and tumor/node/metastasis provided a better model for predicting long-term PFS. Additionally, high SCNA may activate the cell cycle pathway and induce tumorigenesis.

**Conclusions:**

SCNAs may be used to predict PFS in patients with early-stage LUAD with radiotherapy, in combination with TNM, with the aim of predicting long-term PFS. Therefore, SCNAs are a novel predictive biomarker for radiotherapy in patients with LUAD.

## Introduction

Lung cancer is the most common cause of cancer-related deaths worldwide, and non-small cell lung cancer (NSCLC) accounts for more than 80% of diagnosed lung cancers^1^. Radiotherapy is a useful and commonly used therapeutic tool to achieve local tumor control while limiting damage to the surrounding tissues, and it plays an important role in the treatment of lung cancer^2,3^. However, only a subset of patients benefit from radiotherapy, and understanding the molecular determinants of the response to cancer therapy is a pivotal challenge in cancer oncology^4^. In patients with breast cancer receiving adjuvant radiotherapy, circulating tumor cell status is an important clinical indicator predicting benefit from radiotherapy^5^. A study analyzing molecular predictive markers in sarcoma has found significant differences in gene signatures between poor and good responders to radiotherapy^6^.

Somatic copy number alteration (SCNA), known as aneuploidy, is common to many cancers and is responsible for a large proportion of cancer genome alterations^7,8^. SCNA occurs during tumorigenesis, tumor progression, and recurrence, and is a biomarker of prostate cancer response to chemotherapy, melanoma, and lung cancer response to immunotherapy^8–10^. A recent study has found that SCNAs can be used to monitor tumorigenesis in the neoplastic precursor lesion Barrett’s esophagus, and can distinguish progressive from stable disease before histopathological transformation^11^. SCNAs have also been reported to have predictive value in cancer. Recently, the predictive potential of SCNAs has been validated in immunotherapy. For example, in patients with NSCLC treated with anti-programmed cell death (ligand) 1 [PD-(L)1] therapy, SCNAs are lower in patients with durable clinical benefit than in those with nondurable benefit; among patients with melanoma treated with anti-cytotoxic T-lymphocyte associated protein 4 (CTLA-4), those with low SCNA experience longer survival than those with high SCNA^12^. SCNAs are a prognostic factor for prostate cancer-specific death^9^. In both primary and metastatic prostate cancer, SCNAs are a biomarker predictive of overall survival (OS). These observations prompted us to explore whether SCNAs might predict the response of patients with NSCLC to radiotherapy.

The aim of this study was to examine SCNA status in NSCLC and to investigate potential associations with patient survival and the underlying mechanism. We analyzed OS and progression-free survival (PFS) in patients with NSCLC treated with radiotherapy to better understand the influence of SCNA on patients benefiting from radiotherapy.

## Materials and methods

### Patients and tissue samples

Data from 20 patients with a histological diagnosis of LUAD who had received radical (R0) resection at the Tianjin Medical University Cancer Institute and Hospital, between January 2013 and December 2013, were retrospectively collected. All patients underwent radical resection after radiotherapy with 40 Gy/20 f. Detailed patient data are shown in **Table 1**. Experiments on human participants followed the Helsinki Declaration (as revised in 2013). The study was approved by the Ethics Committee of the Tianjin Medical University Cancer Institute and Hospital (Approval No. bc2021077).

**Table 1:** Clinical characteristics of patients with LUAD undergoing radiotherapy (*n* = 20)

Characteristics	LUAD (*n* = 20)
Gender, *n* (%)	
Male	9 (45.00%)
Female	11 (55.00%)
Age, y, *n* (%)	
< 60	9 (45.00%)
≥ 60	11 (55.00%)
Tumor stage, *n* (%)	
I	5 (25.00%)
II	7 (35.00%)
III	7 (35.00%)
IV	1 (5.00%)
Progression, *n* (%)	
No	3 (15.00%)
Yes	17 (85.00%)
Survival, *n* (%)	
No	9 (45.00%)
Yes	10 (50.00%)
NA	1 (5.00%)

### Infinium Asian Screening Array (ASA)

The array was built by using an East Asian reference panel containing 9,000 whole-genome sequences. All DNA samples were extracted with DNA-extraction kits (Tiangen Biotech). Samples were genotyped with the Infinium Asian Screening Array (Illumina) according to the manufacturer’s specifications. The genotyping module of Genomestudio v2.1 (Illumina) was used to call the genotypes.

### The Cancer Genome Atlas (TCGA)

Data from TCGA (https://portal.gdc.cancer.gov/), an online freely accessible database were downloaded. SCNA data were acquired from a study by Thorsson et al. analyzing the number of segments, which represented copy number alterations^13^. From these data, we selected data for 486 patients with NSCLC who received radiotherapy.

### Building and validating a predictive nomogram

Nomograms are widely used to predict prognosis^14,15^. All independent prognostic factors identified by logistic analysis were included to develop a nomogram to assess the probability of 1-, 3-, and 5-year PFS in LUAD. Validation of the nomogram was examined by discrimination and calibration. The concordance index (C-index) was calculated to evaluate the discrimination of the nomogram. The calibration curve was plotted to explore the nomogram’s predicted probabilities *vs.* the observed probabilities.

### Gene set enrichment analyses (GSEA)

To elucidate the potential molecular mechanisms underlying SCNAs we performed GSEA^16^ with Sangerbox tools, a free online platform for data analysis (http://www.sangerbox.com/tool), to explore Kyoto Encyclopedia of Genes and Genomes pathways enriched in low- and high-SCNA samples. The statistical parameters *P* < 0.01, false discovery rate q < 0.05, and |NES| > 1 were in accordance with the inclusion criteria and were considered to indicate statistical significance.

### RT-qPCR

Total RNA was isolated from patients with TRIzol reagent and reverse transcribed into cDNA (GoScript™ Reverse Transcription System, USA). The mRNA expression of pathway-related genes was examined by RT-qPCR. The RT-qPCR primers are provided in **Supplementary Table S1**.

### Statistical analysis

Survival curves were determined by using the Kaplan-Meier method, and differences in PFS and OS among groups were assessed with the log-rank test. Cox proportional model analysis was performed through univariate and multivariable analyses. All statistical analyses were conducted in R software (version 3.5.3).

## Results

### SCNA predicts survival of patients with LUAD treated with radiotherapy

**Figure 1:**
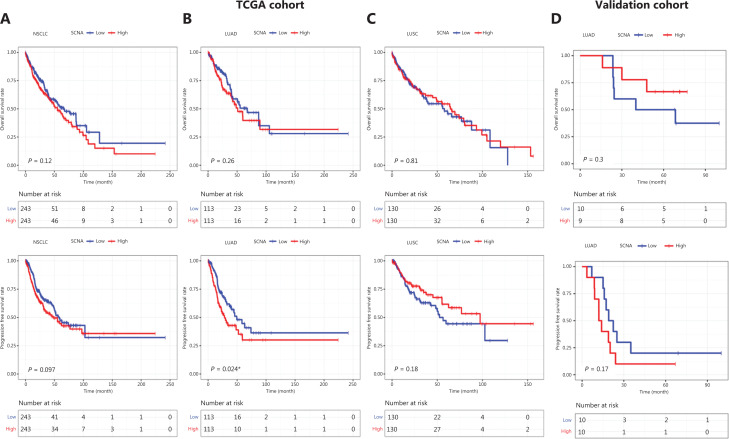
Survival analysis with respect to SCNAs in patients with NSCLC undergoing radiotherapy from TCGA and validation cohort. (A) OS and PFS in patients with NSCLC undergoing radiotherapy from TCGA cohort. (B) OS and PFS in patients with LUAD from TCGA cohort. (C) OS and PFS in patients with LUSC from TCGA cohort. (D) OS and PFS in patients with LUAD from the validation cohort. *P < 0.05.

To understand the influence of SCNA on radiotherapy, we analyzed SCNA data as well as clinical information obtained from TCGA database. Among this cohort, 226 patients with LUAD and 260 patients with lung squamous carcinoma (LUSC) received radiotherapy (**Table 2**). To investigate the prognostic value of SCNA in NSCLC with radiotherapy, we grouped the patients into low- and high-SCNA groups on the basis of the median level in the cohort, in accordance with methods from published studies^9^. As shown in **Figure 1A**, SCNAs were not associated with OS (*P* = 0.12) or PFS (*P* = 0.097) in the full cohort. However, several studies have reported copy number analyses in patients with lung cancer showing that some alterations are common across lung cancers, whereas others vary among specific histologic subtypes^17,18^. To determine whether SCNAs have prognostic value in specific subtypes, we performed separate survival analyses for LUAD and LUSC. In patients with LUAD receiving radiotherapy from TCGA (RT-cohort), no difference in OS was found between low SCNA and high SCNA levels (*P* = 0.26, **Figure 1B**). However, the SCNA level was significantly associated with PFS (*P* = 0.024). In the RT-cohort, a high SCNA level was associated with greater progression. However, in patients with LUSC, no difference in OS (*P* = 0.81) or PFS (*P* = 0.18) was found between low and high SCNA levels (**Figure 1C**). To validate the prognostic ability of SCNA, we collected 20 patients with lung LUAD who received radiotherapy as a control group. In the validation cohort, we observed a trend in which patients with lower SCNA had longer PFS and OS (*P* = 0.17, *P* = 0.3, respectively; **Figure 1D**).

**Table 2:** Clinical characteristics of patients undergoing radiotherapy

Characteristics	Total (*n* = 486)	LUAD (*n* = 226)	LUSC (*n* = 260)
Gender, *n* (%)			
Male	185 (38.07%)	109 (48.23%)	192 (73.85%)
Female	301 (61.93%)	117 (51.77%)	68 (26.15%)
Age, y, *n* (%)			
< 60	103 (21.19%)	60 (26.55%)	43 (16.54%)
≥ 60	374 (76.96%)	161 (71.24%)	213 (81.92%)
NA	9 (1.85%)	5 (2.21%)	4 (1.54%)
Tumor stage, *n* (%)			
I	258 (53.09%)	133 (58.85%)	125 (48.08%)
II	135 (27.78%)	47 (20.80%)	88 (33.84%)
III	70 (14.40%)	29 (12.83%)	41 (15.77%)
IV	16 (3.29%)	12 (5.31%)	4 (1.54%)
NA	7 (1.44%)	5 (2.21%)	2 (0.77%)
NSCLC, *n* (%)			
LUAD	226 (46.50%)	226 (100%)	0
LUSC	260 (53.50%)	0	260 (100%)
Progression, *n* (%)			
No	322 (66.26%)	135 (59.73%)	187 (71.92%)
Yes	260 (33.74%)	91 (40.27%)	73 (28.08%)
Survival, *n* (%)			
No	186 (38.27%)	78 (34.51%)	108 (41.54%)
Yes	300 (61.73%)	148 (65.49%)	152 (58.46%)

**Figure 2:**
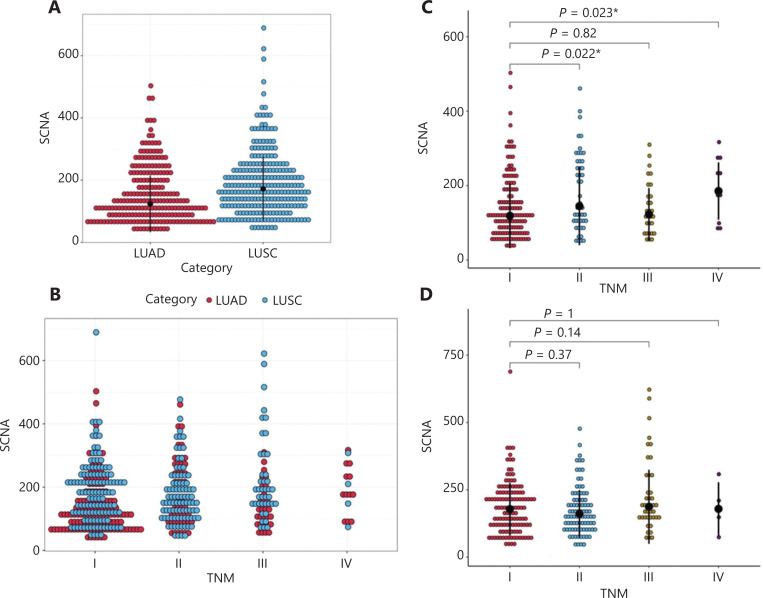
The distribution of SCNAs in different histologic subtypes and TNM stages. (A) Distribution of SCNA levels in LUAD and LUSC. (B) Distribution of SCNA levels in 4 TNM stages. (C) Comparison of SCNA levels among TNM stages I–IV in patients with LUAD. (D) Comparison of SCNA levels among TNM stages I–IV in patients with LUSC. *P < 0.05.

When we compared LUAD with LUSC, we identified significant differences in the following features of SCNAs: the SCNA level (**Figure 2A**) and the distribution of SCNAs in tumor/node/metastasis (TNM) stages I–IV (**Figure 2B**). The distribution of SCNAs in LUSC tended to be more concentrated than that in LUAD. This observation may explain why SCNAs in LUSC did not predict the efficacy of radiotherapy, because the SCNAs did not differentiate between the high and low groups.

### SCNAs are predictive of the survival of patients with early-stage LUAD

**Figure 3:**
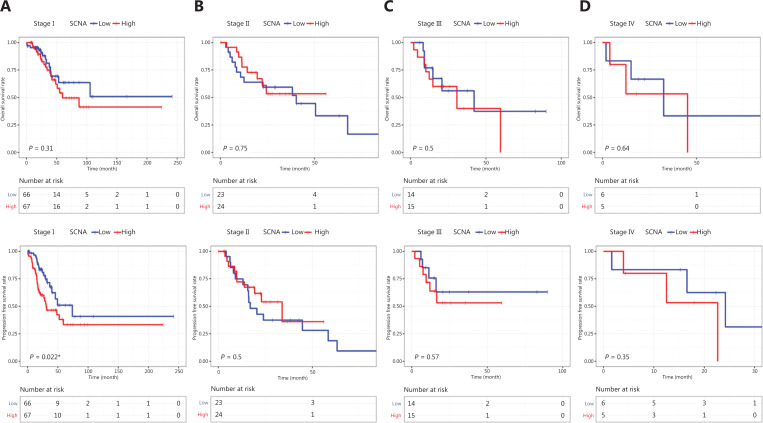
Survival analysis of SCNAs in patients with LUAD undergoing radiotherapy, according to TNM stage. (A) OS and PFS in stage I. (B) OS and PFS in stage II. (C) OS and PFS in stage III. (D) OS and PFS in stage IV. *P < 0.05.

To analyze the predictive ability of SCNAs in patients with LUAD undergoing radiotherapy, we performed Cox proportional hazard regression analyses (**Table 3**). Univariate analyses showed that SCNAs correlated with PFS [hazard ratio (HR) = 2.0948, 95% confidence interval (CI): 1.366–3.211, *P* = 0.0007]. TNM stage I also correlated with PFS (HR = 1.8650, 95% CI: 1.1305–3.077, *P* = 0.0147). In a multivariate analysis, SCNAs remained statistically significant (HR = 2.0756, 95% CI: 1.3264–3.248, *P* = 0.0014). Whether SCNAs might predict the response to radiotherapy in early compared with advanced LUAD was unclear. Therefore, we examined whether survival correlated with SCNA status in patients with LUAD, within each of the TNM stages (I–IV). Patients with stage I LUAD and low SCNA levels had longer PFS than those with high SCNA levels (*P* = 0.022; **Figure 3A**). However, SCNAs were not associated with PFS in patients with stage II (*P* = 0.5), III (*P* = 0.57), or IV (*P* = 0.35; **Figure 3B–3D**) LUAD. In all 4 stages, no significant difference in OS was found between patients with low and high SCNA levels (*P* = 0.31, *P* = 0.75, *P* = 0.5, *P* = 0.64, respectively). We also assessed the distribution of SCNAs across the TNM stages. The SCNA levels in patients with stage II and III LUAD were significantly higher than those in patients with stage I disease, whereas there was no difference in SCNA levels between stages I and III (**Figure 2C**). These results confirmed that SCNA levels were lower in stage I than in II and III stage LUAD Our results thus indicated a difference in SCNA levels between early and advanced LUAD. In LUSC, no significant difference in SCNA levels was found among the 4 stages (**Figure 2D**). Together, these results also suggested the prognostic potential of SCNAs in early-stage LUAD.

**Table 3:** Factors associated with PFS in patients with LUAD undergoing radiotherapy (*n* = 216)

Characteristics	HR (95% CI)	*P* value
Univariate analysis		
Gender (male *vs.* female)	0.8646 (0.5654–1.322)	0.5020
Age (< 60 *vs.* ≥ 60)	1.2771 (0.767–2.126)	0.3470
SCNA (low *vs.* high)	2.0948 (1.366–3.211)	0.0007*
TNM (I *vs*. II)	1.8650 (1.1305–3.077)	0.0147*
TNM (I *vs*. III)	1.2912 (0.6514–2.559)	0.4642
TNM (I *vs*. IV)	2.2676 (0.9634–5.337)	0.0608
Multivariate analysis		
Gender (male *vs*. female)	0.8253 (0.5325–1.279)	0.3902
Age (< 60 *vs*. ≥ 60)	1.4474 (0.8628–2.428)	0.1613
SCNA (low *vs*. high)	2.0756 (1.3264–3.248)	0.0014*
TNM (I *vs*. II)	1.5938 (0.9552–2.659)	0.0743
TNM (I *vs*. III)	1.3074 (0.6570–2.602)	0.4452
TNM (I *vs*. IVa)	1.7708 (0.7350–4.266)	0.2028

*P < 0.05 was considered significant.

### Prognostic nomogram

**Figure 4:**
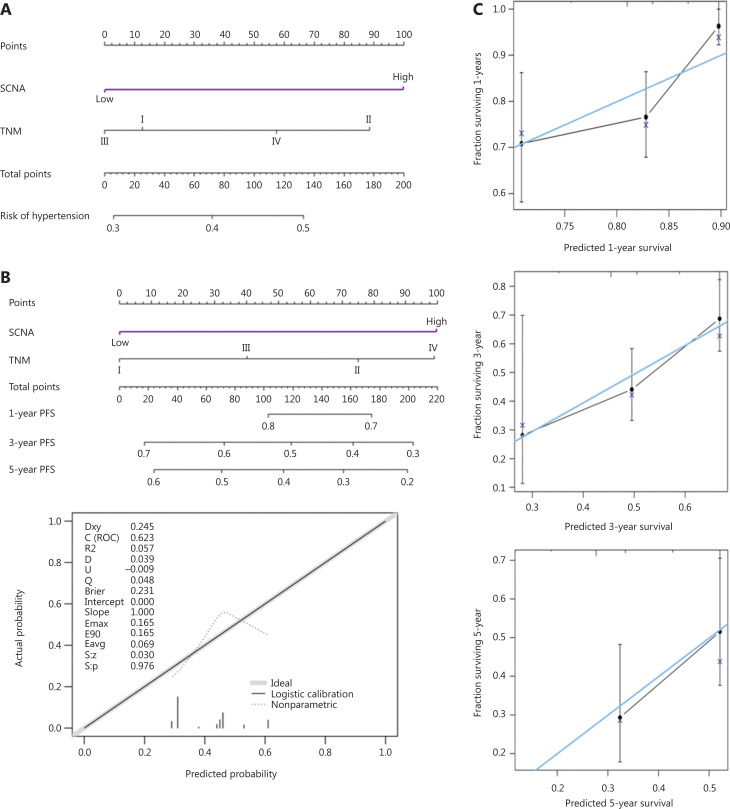
Prognostic nomogram for patients with LUAD undergoing radiotherapy. (A) Nomogram showing the assessment of PFS with SCNAs and TNM. (B) Nomogram for predicting 1-year, 3-year, and 5-year PFS in the RT-LUAD cohort. Calibration plot showing the AUC of the predictive model for PFS. (C) Calibration curve for predicting PFS at 1 year, 3 years, and 5 years in patients with LUAD.

We selected 2 independent prognostic factors, SCNAs and TNM, as variables to develop a nomogram for assessing the survival of patients with LUAD undergoing radiotherapy (**Figure 4A**). Our analyses suggested that 1-, 3-, and 5-year PFS probabilities could be successfully calculated by using these nomograms (**Figure 4B**). The C-index of the nomogram for SCNAs and TNM was 0.623, and the calibration curve displayed good agreement between the probability scores of the 3- and 5-year PFS compared with that of the 1-year PFS (**Figure 4A and 4B**). These findings support the prognostic potential of SCNAs in patients with LUAD undergoing radiotherapy, particularly with respect to long-term PFS.

### Significant pathways influenced by SCNAs

**Figure 5:**
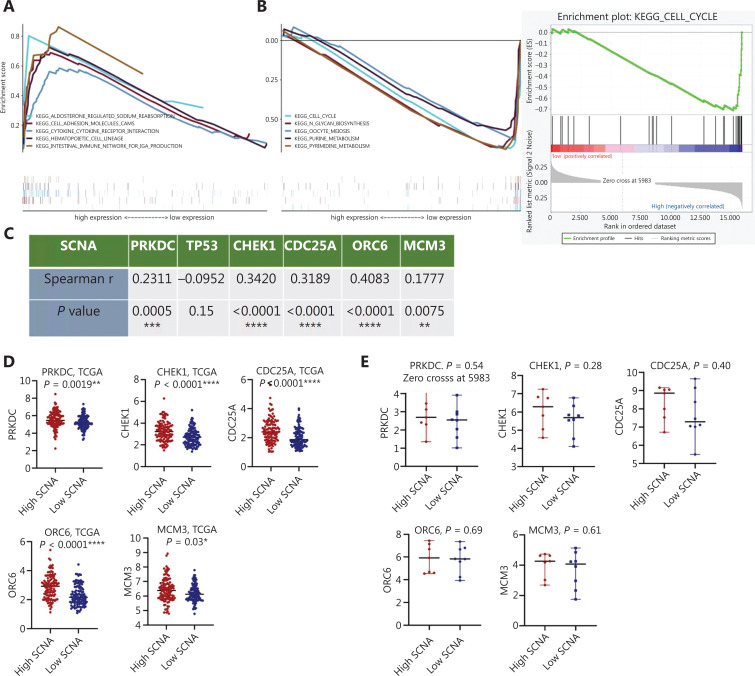
Underlying pathways in patients with LUAD with high *vs.* low SCNA levels. (A & B) GSEA used to identify KEGG in the low SCNA group and high SCNA group, respectively. According to the false discovery rate q-value, the 5 most significant pathways are shown. (C) Association of the key genes in the cell cycle pathway with SCNAs in the low and high SCNA groups in TCGA cohort. (D) Expression levels of the key genes in the cell cycle pathway (PRKDC, CHEK1, CDC25A, ORC6, and MCM3) in the low and high SCNA groups in TCGA cohort. (E) Expression levels of the key genes in the cell cycle pathway (PRKDC, CHEK1, CDC25A, ORC6, and MCM3) in the low and high SCNA groups, detected in 15 patients with LUAD by RT-qPCR. Significance is given as *P < 0.05, **P < 0.01, ***P < 0.001 and ****P < 0.0001.

To further investigate the potential functions of SCNAs, we performed GSEA to investigate the molecular mechanisms underlying the SCNAs and to identify the pathways involved. In the low-SCNA group, the 5 most significantly enriched pathways were “aldosterone regulated sodium reabsorption”, “cell adhesion molecules”, “cytokine-cytokine receptor interaction”, “hematopoietic cell lineage”, and “intestinal immune network for immunoglobulin (Ig)A production” (**Figure 5A**). In the high-SCNA group, the 5 most significantly enriched pathways were “cell cycle”, “N-glycan biosynthesis”, “oocyte meiosis”, “purine metabolism”, and “pyrimidine metabolism” (**Figure 5B**). Considering that high SCNA was significantly correlated with poor survival, we focused on verifying the term “cell cycle”, which was enriched in the high SCNA group. First, GSEA of TCGA showed that the key genes in the cell cycle pathway were PRKDC, CHEK1, CDC25A, ORC6, and MCM3; these genes were positively associated with SCNAs (**Figure 5C**). Next, we compared the expression of these genes in the low and high SCNA groups from TCGA, and found that these genes were significantly upregulated in the high SCNA group (*P* < 0.05, **Figure 5D**). We then validated the results in 15 patients with LUAD by RT-qPCR; the results were consistent with the TCGA analysis findings but were not significant (**Figure 5E**). Collectively, high SCNA levels elevate the expression of PRKDC, CHEK1, CDC25A, ORC6, and MCM3, thereby activating the cell cycle pathway and inducing tumorigenesis.

## Discussion

SCNAs are widespread in cancers and correlated with tumorigenesis^8,10^. Understanding how SCNAs drive tumorigenesis and metastasis has been a major research area in precision medicine-based oncology. Remarkable technological advances have allowed researchers to acquire and quantify SCNAs. Whole-exome sequencing (WES) of bulk tumor samples has recently become more common, and MSK-IMPACT and Holistic allele-specific tumor copy-number heterogeneity sequencing have also been developed and applied to acquire SCNAs^19–22^. In this study, we analyzed the clinical benefits of radiotherapy for patients with LUAD with low SCNA levels. Additionally, we provided evidence supporting an association between the expression of key genes of the cell cycle pathway and SCNA levels.

We first analyzed the association between SCNAs and response to radiotherapy in patients with NSCLC and found that patients with high SCNA levels had poorer survival. Previously reported flow cytometry analyses of the tumor aneuploidy and ploidy response to radiotherapy have revealed that the high S-phase fraction, which is critical to cell proliferation, is significantly correlated with aneuploidy^23–25^. In addition, copy number gains of oncogenes predict poor clinical outcomes in radiotherapy^26,27^. An important strength of this study is the exploration of the predictive value of SCNAs in different TNM stages. In LUAD, we observed that the SCNA levels in stage III and IV were significantly higher those in stage I. A recent study has also reported a general trend of low TNM stages and histological pathologic subcategories with lower SCNA levels in LUAD^28^. In LUAD, circulating tumor cells with high ploidy have been demonstrated to be associated with tumor resistance and relapse, and in patients in advanced cancer stages, pentaploidy is prevalent^29,30^. The influence of SCNAs on cancer development may be associated with genomic instability. DNA mismatch repair (MMR) proteins, which correct base incorporation errors, are critical for maintaining genomic stability^31^. Inactivation of MMR leads to SCNA^31,32^. Therefore, MMR inactivation can directly lead to SCNA and contribute to cancer progression. Additionally, SCNAs are superior in predicting PFS in patients with early-stage rather than late-stage LUAD. Advanced cancer is a heterogeneous disease, and excessive levels of SCNAs cause heterogeneous karyotypes to lose their selective advantage^33^. Therefore, the predictive value of SCNAs is not clear in late-stage LUAD, and predicting the survival of patients with late-stage LUAD may be more significant by combining SCNAs with heterogeneity. Collectively, the potential reason that SCNAs contribute to cancer progression and predict prognosis for early-stage patients with LUAD may be correlated with MMR and heterogeneity. Cox proportional regression analyses and nomograms showed that SCNAs were a reliable prognostic indicator for lung cancer patients. Notably, the nomogram curve combining SCNAs and TNM to predict 3- or 5-year PFS provided a better fit of the predictive probabilities than the 1-year PFS nomogram. This observation also suggests a possible role of SCNAs in the recurrence and progression of cancer.

Recently, the predictive potential of SCNAs has been validated in immunotherapy. Davoli et al. selected 2 clinical trials of patients with metastatic melanoma treated with immune checkpoint blockade, and their results revealed that aneuploidy is inversely correlated with patient survival^8^. Kim et al.^10^ have performed WES to acquire SCNAs, and shown that SCNAs improve the accuracy of predicting the immune checkpoint blockade response in lung cancer. SCNAs have been correlated with tumor immunity and the immunotherapy response, and may be considered prognostic biomarkers for cancer immunotherapy^34^. An immune-cold subtype with the least amount of lymphocyte infiltration has been found to have a high SCNA level^35^. Therefore, the relationship between SCNAs and the immune microenvironment may be an indirect reason for the association between high SCNA levels and poorer survival.

In addition to the total SCNAs in patients affecting survival, genes with altered copy numbers simultaneously have the potential to confer multiple phenotypes and response to therapy. The genomic landscape has revealed that copy-number amplified genes within SCNAs may be critical drivers of cancer progression^36^. For example, in predicting tumor shrinkage and progression, HER2 SCNA has been found to perform better than plasma carcinoembryonic antigen levels in gastric cancer^37^. Because most alterations in gene expression are significantly correlated with their CNVs, Shao et al. have analyzed TCGA database and found that genes with SCNA show alterations in downstream gene expression and transcription, particularly for oncogenes and tumor suppressor genes^38^. Moreover, amplification of RALA may affect the biology of EGFR mutant cancer^39^. Cancer is driven by multiple types of genetic alterations, which range in size from mutations to SCNAs^40^. How SCNAs contribute to tumorigenesis and progression must be further investigated.

When we investigated the underlying molecular mechanism of SCNAs in patients with LUAD undergoing radiotherapy, we identified that the cell cycle pathway was enriched in the high-SCNA group. Through TCGA and RT-qPCR analysis, we found that patients with high SCNA levels may have greater expression of key cell cycle genes, thereby inducing tumorigenesis. Because the sample size was limited, more samples and experiments are needed to further verify this signaling pathway in future studies.

## Conclusions

SCNAs are a better biomarker of PFS in LUAD than LUSC in patients undergoing radiotherapy. They may be used to predict PFS in patients with early-stage LUAD undergoing radiotherapy, in combination with TNM, with the aim of predicting long-term PFS. Tumors with high SCNA showed elevated expression of genes in the cell cycle pathway, thus simultaneously promoting tumor proliferation and being associated with poorer prognosis. Therefore, SCNAs may serve as a biomarker indicating radiotherapy prognosis and may guide radiotherapy decisions for patients. Further studies on the mechanisms of SCNAs in cancer progression are needed in the future.
