# Supplementary material for: Somatic copy number alterations are predictive of progression-free survival in patients with lung adenocarcinoma undergoing radiotherapy
Source: Cancer Biol Med. 2021 Aug 27;19(5):685–95. doi: 10.20892/j.issn.2095-3941.2020.0728 (PMC9196051; doi:10.20892/j.issn.2095-3941.2020.0728)
Supplement: Supplementary file 1 [file cbm-19-685-s001.pdf]

Supplementary material

Table S1 The RT-qPCR primers

PRKDC	Forward	TTCTCAAGGAAGAAGGTGTCTC
	Reverse	CAATGAACGTGTTGTAGCACTC
CHEK1	Forward	CTCAAGTTTTGGCGGGAAAAG
	Reverse	AAGTTGAACTTCTCCATAGGCA
CDC25A	Forward	CGAGTCAACAGATTCAGGTTTC
	Reverse	CGATGAGCTGAAAGATGTCATG
ORC6	Forward	GAGGAAAGCAGAGGAGTACTTG
	Reverse	CAGCTCTGATATGTCTCCTTGT
MCM3	Forward	CTTCTAATAGGAGACCCATCCG
	Reverse	AATTCATCAATGCAAACCACGC
